# Immunological characterization of pleural effusions in pediatric patients

**DOI:** 10.3389/fimmu.2024.1506073

**Published:** 2024-12-16

**Authors:** Luca Flögel, Elisabeth Kaiser, Muriel Charlotte Hans, Sybelle Goedicke-Fritz, Michelle Bous, Hashim Abdul-Khaliq, Martin Poryo, Michael Zemlin, Regine Weber

**Affiliations:** ^1^ Department of General Pediatrics and Neonatology, Saarland University, Campus Homburg, Homburg, Germany; ^2^ Department of Pediatric Cardiology, Saarland University Medical Center, Homburg, Germany

**Keywords:** pleural effusion, T cells immunology differentiation, neonates, cardiac surgery, pediatrics

## Abstract

**Background:**

The pleural cavity represents a unique immunological compartment that can mount inflammatory reactions during infections, after surgery and in chronic immunological diseases. The connection between systemic immune reactions in the blood and local immune reactions in pleural effusions remains unclear. This study provides the first comprehensive immunological characterization of paired blood and pleural effusion samples, utilizing combined cell and cytokine analyses in pediatric patients undergoing cardiac surgery.

**Methods:**

In 30 pediatric patients (median age: 22 months) with pleural effusion after cardiac surgery for congenital heart defects, corresponding peripheral blood and pleural effusion samples were analyzed for their immune response. We used flow cytometry and multiplex immunoassays to quantify 14 T cell subpopulations and 12 T cell associated cytokines in each biosample.

**Results:**

IL-6, IL-8, IL-10, TNF (p<0.0001) levels were significantly higher in pleural effusion compared to plasma. In contrast, IFN-γ, GM-CSF, IL-17A levels were lower in pleural effusion than in plasma (p ≤ 0.0005). In comparison to peripheral blood, there was a significantly higher proportion of T helper cells 1 (T_h_1, p=0.0023), T helper cells 17 (T_h_17, p=0.0334) and memory effector cytotoxic T cells (CD3^+^CD8^+^CD45RO^+^CD62L^-^, p=0.0449) in pleural effusion and the same trend was observed for memory effector T_h_ cells (CD3^+^CD4^+^CD45RO^+^CD62L^-^, p=0.0633) and double-negative T cells (CD3^+^CD4^-^CD8^-^) (p=0.1085). Naïve T_h_ cells (CD3^+^CD4^+^CD45RO^-^CD62L^+^) and naïve cytotoxic T cells (CD3^+^CD8^+^CD45RO^-^CD62L^+^) were slightly reduced in pleural effusion compared to peripheral blood (not significant).

**Conclusion:**

Immunological factors in pleural effusions differed significantly from the corresponding blood samples in pediatric patients after cardiac surgery. The results suggest localized production of specific cytokines within the pleural space, while the distribution of other cytokines in pleural effusions appears to be more reflective of the systemic immune response. We found evidence that on the cellular level, the surface marker CD62L may play a key role in navigating T cells between the blood and pleural effusion. This study confirms that the pleural cavity harbors a unique lymphatic compartment, the analysis of which may be useful for both diagnostic and therapeutic purposes.

## 1 Introduction

Pleural effusions (PE) represent the accumulation of fluid in the pleural cavity, commonly observed in pediatric patients, particularly newborns (1–3). Pleural effusions occur as a complication after cardiac surgery in pediatric patients with an incidence of up to one third (4). Clinical outcomes of PE vary widely, ranging from an asymptomatic course and spontaneous recovery to life-threatening compression of the lungs, requiring emergency drainage (5–7). Chylothorax, a subtype of PE caused by damage to the thoracic duct especially during cardiac surgery, is the most common subtype of PE in newborns (1, 8, 9). It is characterized by the loss of considerable amounts of fats, vitamins, antibodies and lymphocytes. In consequence, chylothorax can lead to secondary immune deficiency, failure to thrive and increased risk of sepsis (10–16).

Immune cells and cytokines are lost into the effusion and during puncture, which might lead to dysregulation of the immune system. Patterns of lymphocyte subpopulations and cytokine expression can be useful biomarkers to distinguish between various causes of inflammation (17–21). Hitherto, immunological characterizations of PE samples were mostly focused on oncological diseases or infections and were limited to a very small number of cases or to a few cell populations. Usually, the cytokines that are secreted by these cells, were not studied. For example, regulatory T cells (T_reg_), monocytes, IL-17A and IFN-γ have been described as biomarker candidates, but only for the diagnosis of malignant (22–27) or infectious PE (28–31). In a small number of pediatric patients with a chylothorax after cardiac surgery, different expression levels of individual T cell subpopulations were detected when comparing peripheral blood and PE (32, 33).

Immunological changes in the patient’s peripheral blood are known to occur after cardiac surgery (34–36). However, to date, PE associated with cardiac surgery is poorly understood since the lymphocyte subpopulations and cytokine patterns have not been studied in paired peripheral blood and PE samples (4, 7, 37, 38).

Here, we present the first study investigating 16 T lymphocyte subpopulations and 12 cytokines in both peripheral blood and PE in pediatric patients following cardiac surgery. The study focuses on characterization of T cell immunity and also considers innate and adaptive immune responses. Studies demonstrate that the pleural cavity represents a unique immunological compartment, which is in close interaction with peripheral blood. Immunological biomarkers in the PE may be of diagnostic value to monitor and control individualized therapy.

## 2 Materials and methods

### 2.1 Study design and data collection

Thirty patients were recruited between June 2021 and September 2022 at the Department of Pediatrics, at the Saarland University Medical Center (Homburg, Germany) after written informed consent which was obtained from the parents. The study was designed in accordance to the Declaration of Helsinki, and approved by the Ethics committee of the Medical Association of Saarland (study number 57/20, date of approval 26^th^ April 2021). The inclusion criteria of the study were age between 0 and 18 years and presence of a PE after cardiac surgery. All documentation and data collected were pseudonymized. Both, peripheral blood and PE samples were collected simultaneously and prepared for final analysis on the same day.

### 2.2 Patients characteristics

A total of 30 patients (21 male, 9 female) with a median age of 22 months were included in the study. All patients suffered from congenital heart defects, which were treated by cardiac surgery. Notably, combinations of several heart defects were often present and combined surgeries were performed accordingly. Detailed patient’s characteristics are given in **Table 1**.

**Table 1:** Patients characteristics.

		Total	Female	Male
Demography (absolute number)		30	9	21
Age (months)	Median	22	7	50
	IQR	3.8 – 101.8	3.0 – 54.0	3.5 – 125.5
	Range	0.0 – 225.0	0.0 – 120.0	0.0 – 225.0
Congenital heart defects indicating surgery (absolute number)	VSD	10	4	6
	PDA	6	1	5
	ASD type 2	4	2	2
	Aortic valve stenosis	4	0	4
	CoA	3	1	2
	Others*	9	3	6
Type of surgery (absolute number)	VSD closure	5	1	4
	Pulmonary artery banding	5	2	3
	PDA closure	6	1	5
	ASD closure	4	2	2
	Aortic valve reconstruction	4	0	4
	CoA repair	3	1	2
	Others**	9	3	6
Duration of surgery (minutes)	Median	127.0	173.5	125.0
	IQR	98 - 167	98.25 – 204.5	98.0 – 165.0
	Range	21.0 – 220.0	34.0 – 220.0	21.0 – 213.0
Cardiopulmonary bypass (absolute number)		16	5	11
Duration of cardiopulmonary bypass (minutes)	Median	71.0	105.0	60.0
	IQR	53.3 – 107.3	62.0 – 110.5	53.0 – 87.0
	Range	40.0 – 139.0	47.0 – 113.0	40.0 – 139.0
Period until collection of biosamples after surgery (days)	Median	2.0	4.0	2.0
	IQR	1.0 - 3.5	2.0 – 5.0	1.0 – 3.0
	Range	1.0 - 8.0	2.0 – 8.0	1.0 – 4.0

Age, congenital heart defects indicating surgery, characteristics of surgery and period until collection of biosamples after surgery classified according to sex. IQR, interquartile range; VSD, Ventricular septal defect; ASD, Atrial septal defect; PDA, Patent ductus arteriosus; PFO, Patent foramen ovale; CoA, Coarctation of the aorta. * = Mitral valve regurgitation (n=1), tricuspid valve regurgitation (n=1), aortic valve stenosis (n=1), pulmonary artery stenosis (n=1), hypoplastic left heart syndrome (n=2), Tetralogy of Fallot (n=1), hypoplastic aortic arch (n=1) and/or coronary artery anomaly (n=1). ** = Mitral valve repair (n=1), tricuspid valve repair (n=1), Ross procedure (n=1), surgical repair of pulmonary artery stenosis (n=1) Glenn procedure (n=1), Fontan procedure (n=1), surgical repair of Tetralogy of Fallot (n=1), aortic arch reconstruction with patch augmentation (n=1) or reimplantation of the left anterior descending artery (n=1).

### 2.3 Sample collection and preparation

#### 2.3.1 Peripheral blood samples

Peripheral blood mononuclear cells (PBMC) and plasma were obtained from EDTA blood samples during routine diagnostic blood drawls. Plasma was obtained by centrifugation of the whole blood sample (400 g for 30 min) prior to cryopreservation at -80°C. PBMC were isolated by density gradient centrifugation using the Lymphocyte Separation Medium 1.077, FicoLite-H (#Linaris biological products, Dossenheim, Germany). The PBMC fraction was washed twice using Dulbecco’s phosphate-buffered saline (D-PBS, #D8537, Sigma Aldrich, Steinheim, Germany) by centrifugation at 400 g for 10 min. Cells were counted and viability was calculated by acridine orange and propidium iodide staining (LUNA-FL™ Automated Fluorescence Cell Counter and AO/PI staining, #F23001, Logos Biosystems, Dongan-gu Anyang-si, Gyeonggi-do, South Korea) prior to resuspension in fetal bovine serum (FBS, Thermo Fisher Scientific, Waltham, MA, USA) + 10% dimethylsulfoxid (DMSO, #D8418 Sigma Aldrich by Merck, Steinheim, Germany). The samples were prepared for cryopreservation at −80°C using a CellCamper^®^.

#### 2.3.2 Pleural effusion samples

PE samples were obtained using the thoracic drainage system Atmos^®^ C 051 Thorax (ATMOS MedizinTechnik GmbH & Co. KG, Lenzkirch, Germany). The components of the PE samples were separated by centrifugation (16,100 g for 15 min). The cell-free supernatant was separated and stored at -80°C for further analysis. The cell fraction (pleural effusion cells, PEC) was washed twice with D-PBS and centrifuged (200 g for 10 min). Cell counting and preparation for cryopreservation was performed analogous to PBMC processing.

### 2.4 Flow Cytometry

Cryopreserved cells were thawed on the day of the experiment, washed twice with D-PBS (#D8537, Sigma Aldrich, Steinheim, Germany) and centrifuged at 400 g for 10 minutes before determining the number and viability of cells using acridine orange and propidium iodide (AO/PI, #F23001, Logos Biosystems, Dongan-gu Anyang-si, Gyeonggi-do, South Korea) and LUNA-FL™ Automated Fluorescence Cell Counter (Logos Biosystems, Dongan-gu Anyang-si, Gyeonggi-do, South Korea). Staining of dead cells was performed with BD Horizon Fixable Viability Stain 780 (#565388, BD Biosciences, Heidelberg, Germany), followed by washing with BD CellWASH™ (#349524, BD Biosciences, Heidelberg, Germany) and centrifugation (10 minutes, 500 g). Cell pellets were resuspended in an antibody mix (**Supplementary File 4** in **Supplementary Data Sheet 1**) containing BD Horizon™ Brilliant Stain Buffer (#563794, BD Biosciences, Heidelberg, Germany) and BD Pharmingen™ Stain buffer BSA (#554657, BD Biosciences, Heidelberg, Germany). After 30 min of light protected incubation, the remaining erythrocytes were lysed with BD FACS™ Lysing Solution (#349202, BD Biosciences, Heidelberg, Germany) according to the manufacturer’s protocol. Thereafter, the cell suspension was diluted with BD CellWASH™ and centrifuged at 500 g for 10 minutes. Cell pellets resuspended in D-PBS were analyzed using a 3-laser 12-color FACS Celesta flow cytometer (Becton, Dickinson and Company, Heidelberg, Germany). For the quality control, the following approaches were used: The Cytometer Setup and Tracking Module (CS&T, #655051, BD Biosciences, Heidelberg, Germany) was used daily to check and maintain the flow cytometer performance, stability, fluorescence calibrations and reproducibility of the data. To ensure reproducibility of the experiments, Sphero™ Rainbow Calibration Particles (8 Peaks, #559123, BD Biosciences, Heidelberg, Germany) were used with consistent application settings for each acquisition. Compensation settings were calculated using BD™ CompBeads (#552843, BD Biosciences, Heidelberg, Germany). Positive staining and gating strategies were determined by comparing immunostained samples to unstained controls that underwent all procedures except antibody staining, and isotype controls to assess non-specific antibody binding (**Supplementary Files 1**-**3** in **Supplementary Data Sheet 1**). Cell aggregates were excluded from the analysis (FSC-A/FSC-H) and dead cells were identified and excluded from the analysis by staining with BD Horizon Fixable Viability Stain 780. Finally, the lymphocytes were identified according to morphological parameters (FSC-A/SSC-A). Data were acquired with FACSDiva (BD Biosciences, Heidelberg, Germany) and analyzed using FlowJo v10 (BD Biosciences).

### 2.5 BCA protein assay

Bicinchoninic acid (BCA) Protein Assay was used to determine the total protein concentration in plasma and PE supernatant. The assay was performed using the Pierce™ BCA Protein Assay Kit (#23225, Thermo Fisher Scientific™, Waltham, MA, USA) according to the manufacturer’s instructions. The samples (diluted 1:100 with distilled water) and standards with known protein concentrations were measured in triplicates using NanoDrop™ One (Thermo Fisher Scientific™, Waltham, MA, USA) for subsequent determination of the protein concentration of the samples.

### 2.6 Multiplex immunoassay

For simultaneous quantification of multiple cytokines two customizable multiplex immunoassays were employed: The MILLIPLEX^®^ Human High Sensitivity T Cell Mag Panel (#HSTCMAG-28SK, Merck KGaA, Darmstadt, Germany) and the MILLIPLEX^®^ Human Cytokine/Chemokine/Growth Factor Panel A Magnetic Bead Panel (#HCYTA-60K-1, Merck KGaA, Darmstadt, Germany). Plasma and PE samples were thawed at room temperature, vortexed and centrifuged at 1000 g for 10 minutes. Samples, standards (in seven defined concentration levels), and quality controls (in two defined concentrations) were processed in technical triplicates on a 96-well microtiter plate. Antibody-immobilized magnetic microspheres were sonicated, vortexed, pooled, and supplemented with the provided microsphere diluent. Singleplex-microspheres or mixed multiplex microspheres for customized cytokine profiles were added to the samples and incubated overnight at 4°C. Following multiple washing steps, a biotinylated detection antibody cocktail was added and incubated at room temperature and with shaking for an hour, followed by streptavidin-phycoerythrin incubation for 30 min to label multiplexes. After final wash steps, MAGPIX™ Drive Fluid Plus (Luminex Corp., Austin, TX, USA) was added prior to data acquisition on MAGPIX^®^ instruments with xPONENT^®^ Software (Luminex Corp., Austin, TX, USA). Sample values for each cytokine were determined following standard curve calculation by using 4 and 5 parameter logistics weighted curve fitting algorithms with Belysa^®^ version 1.2 (Millipore by Merck, Burlington, MA, USA). Cytokine levels below limits of detection (LoD) were adjusted to half of the minimal detectable concentration (MinDC) specified by the manufacturer of the immunoassay. Cytokine levels above the quantifiable range were adjusted to the highest standard (Std 7).

The following analytes were included for HSTCMAG-28-SK assay to create a custom cytokine profile: Tumor necrosis factor (TNF), granulocyte-macrophage colony-stimulating factor (GM-CSF), interferon-γ (IFN–γ), interleukin-4 (IL-4), interleukin-5 (IL-5), interleukin-6 (IL-6), interleukin-7 (IL-7), interleukin-8 (IL-8), interleukin-10 (IL-10), interleukin-12 subunit p70 (IL-12p70), interleukin-13 (IL-13), interleukin-17A (IL-17A). In addition, IL-6 was measured in PE samples as a singleplex assay using HCYTA-60K. Samples have been prediluted 1:4 in assay buffer prior to HCYTA-60K assay procedure. Quality controls for all analytes met the manufacturer’s specifications with a maximum interassay coefficient of variation of 12%.

The MAGPIX^®^ instrument was calibrated and validated weekly according to the manufacturer’s instructions, using the MAGPIX^®^ calibration kit (MPX-CAL-K25, Luminex Corp., Texas, United States) and MAGPIX^®^ performance verification kit (#MPX-PVER-K25, Luminex Corp., Texas, United States).

### 2.7 Classification of pleural effusion samples

Light’s criteria were used to distinguish the PE samples representing exudates from transudates. According to Light’s criteria a PE is defined as an exudate if at least one of the following criteria is met: First criterion: The quotient of PE total protein and serum total protein is higher than 0.5. Second criterion: The quotient of PE lactate dehydrogenase (LDH) and serum LDH is higher than 0.6. Third criterion: PE LDH is at least 2/3 of the upper limit of normal serum LDH (39, 40). In the present study, we applied the first criterion for the classification of PE, however we applied it to plasma rather than serum.

### 2.8 Statistical analysis

Statistical analysis was performed using Prism 10.1.1 (GraphPad Software LLC, Boston, MA, USA). Quantitative data derived from protein multiplex immunoassay and flow cytometric analyses of cell populations were subjected to the D’Agostino-Pearson test for normal distribution. Data are presented as median, interquartile range (IQR) and individual data points. Flow cytometry data are calculated in relation to the respective parent population, cytokine levels are given as concentration in pg/ml. PE-to-plasma protein ratios are calculated first as the quotient of the total protein level of the cell-free fraction of PE samples and their corresponding plasma samples, and second as specific protein ratios as the quotient of the cytokine level in the pleural effusion and the cytokine level in the corresponding plasma samples. Correlation between T cell populations and cytokines was determined in both blood and pleural effusion. Spearman’s rank correlation was used to determine correlation coefficient (r) and p-value of every combination of T cell population and cytokine. Sample group differences (PEC vs. PBMC) analyzed by flow cytometry as well as patient’s group differences (<12 months vs. >12 months, CPB vs. no-CPB and female vs. male) regarding cytokine expression were assessed using two-tailed Mann-Whitney-U-test. Group differences regarding protein levels (plasma vs. PE) were assessed using two-tailed Wilcoxon matched-paired signed rank for paired data. Confidence level for statistical significance was set at 95%.

## 3 Results

### 3.1 Flow cytometric analysis of PBMC and PEC

#### 3.1.1 Different frequency of T helper subsets in PBMC and PEC

**Figure 1:**
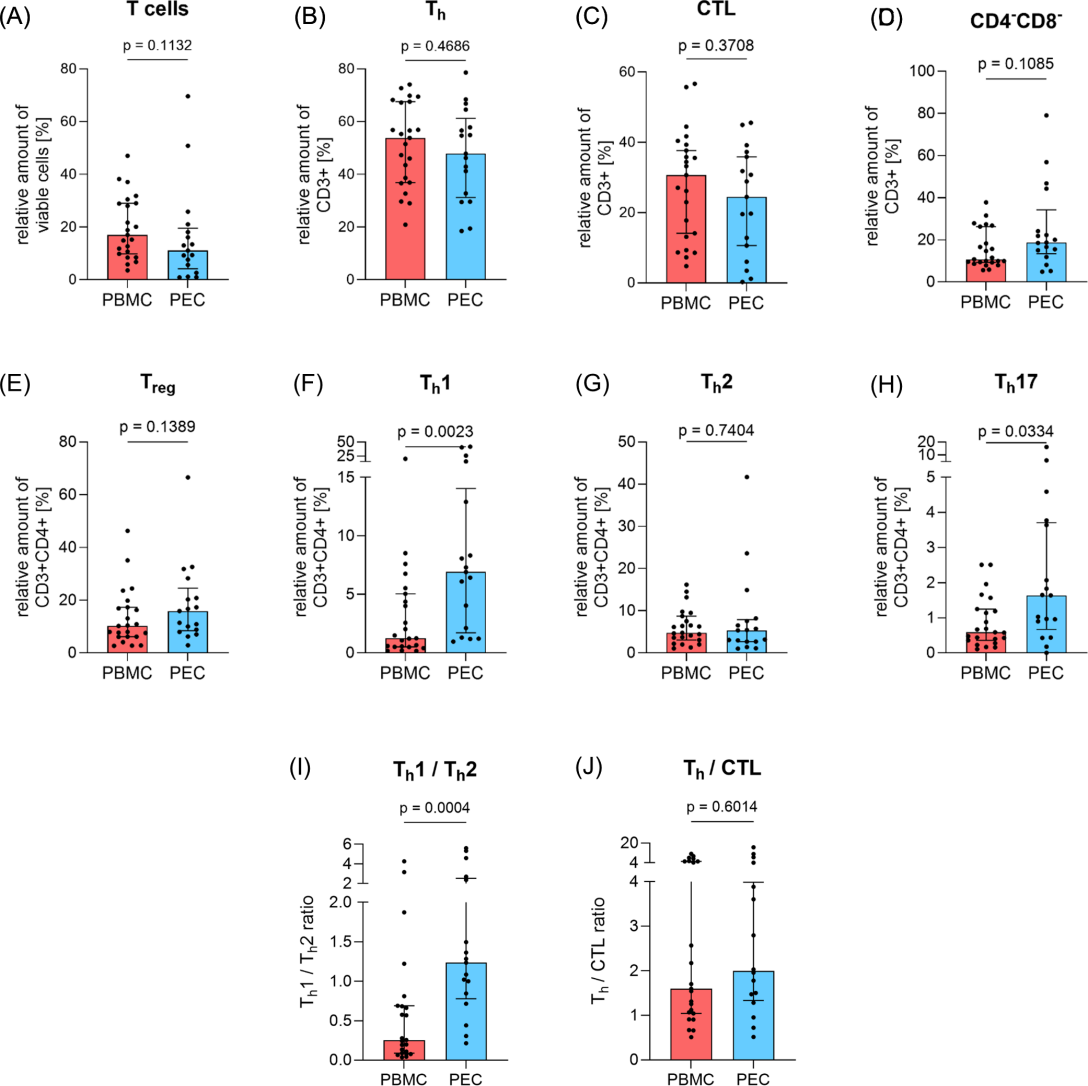
Amount of basic T cell subpopulations in peripheral blood and pleural effusion. The proportion of T cell subpopulations **(A)** total T cells (CD3^+^), **(B)** T helper cells (T_h_, CD3^+^CD4^+^), **(C)** cytotoxic T cells (CTL, CD3^+^CD8^+^), **(D)** double-negative T cells (DN, CD3^+^CD4^-^CD8^-^), **(E)** regulatory T cells (T_reg_, CD3^+^CD4^+^CD25^+^CD127^-^), **(F)** T helper cells 1 (T_h_1, CD3^+^CD4^+^CD183^+^CD196^-^), **(G)** T helper cells 2 (T_h_2, CD3^+^CD4^+^CD194^+^CD294^+^) and **(H)** T helper cells 17 (T_h_17, CD3^+^CD4^+^CD183^-^CD196^+^) in relation to parent cell population was determined by flow cytometry in PBMC (red, n=23) and PEC (blue, n=17). **(I)** shows the T_h_1/T_h_2 ratio and **(J)** the T_h_/CTL ratio in PBMC and PEC. Bars represent median value, error bars represent interquartile range, dots are drawn as individual data points. Group differences were assessed using two-tailed Mann-Whitney-U-test.

The analysis of the corresponding PBMC and PEC using flow cytometry revealed no significant differences in the expression of overall T cell numbers (CD3^+^) although there was a tendency for quantitative reduction of T cells in PEC (**Figure 1A**). The same applied to the expression of T helper cells (T_h_, CD3^+^CD4^+^, **Figure 1B**) and cytotoxic T lymphocytes (CTL, CD3^+^CD8^+^, **Figure 1C**). T_h_ cells predominate CTL in both PBMC and PEC, which is underlined by the determination of the Th/CTL ratio (**Figure 1J**).

Although T_h_ cells tend to be reduced in PEC, a reverse result was observed in T helper cells 1 (T_h_1, CD3^+^CD4^+^CD183^+^CD196^-^, **Figure 1F**) and T helper cells 17 (T_h_17, CD3^+^CD4^+^CD183^-^CD196^+^, **Figure 1H**) as they were significantly elevated in PEC (p=0.0023 and p=0.0334, respectively). However, the proportion of T helper cells 2 (T_h_2, CD3^+^CD4^+^CD194^+^CD294^+^, **Figure 1G**) and regulatory T cells (T_reg_, CD3^+^CD4^+^CD25^+^CD127^-^, **Figure 1E**) showed similar expression in both PBMC and PEC, whereby T_reg_ also tended to be slightly increased in PEC. The T_h_1/T_h_2 ratio (**Figure 1I**) was significantly increased in PEC (p=0.0004).

Double-negative T cells (DN T cells, CD3^+^CD4^-^CD8^-^, **Figure 1D**) showed no significant difference between both compartments, although the relative amount of DN T cells in PE was almost twice as high as in PBMC.

**Figure 2:**
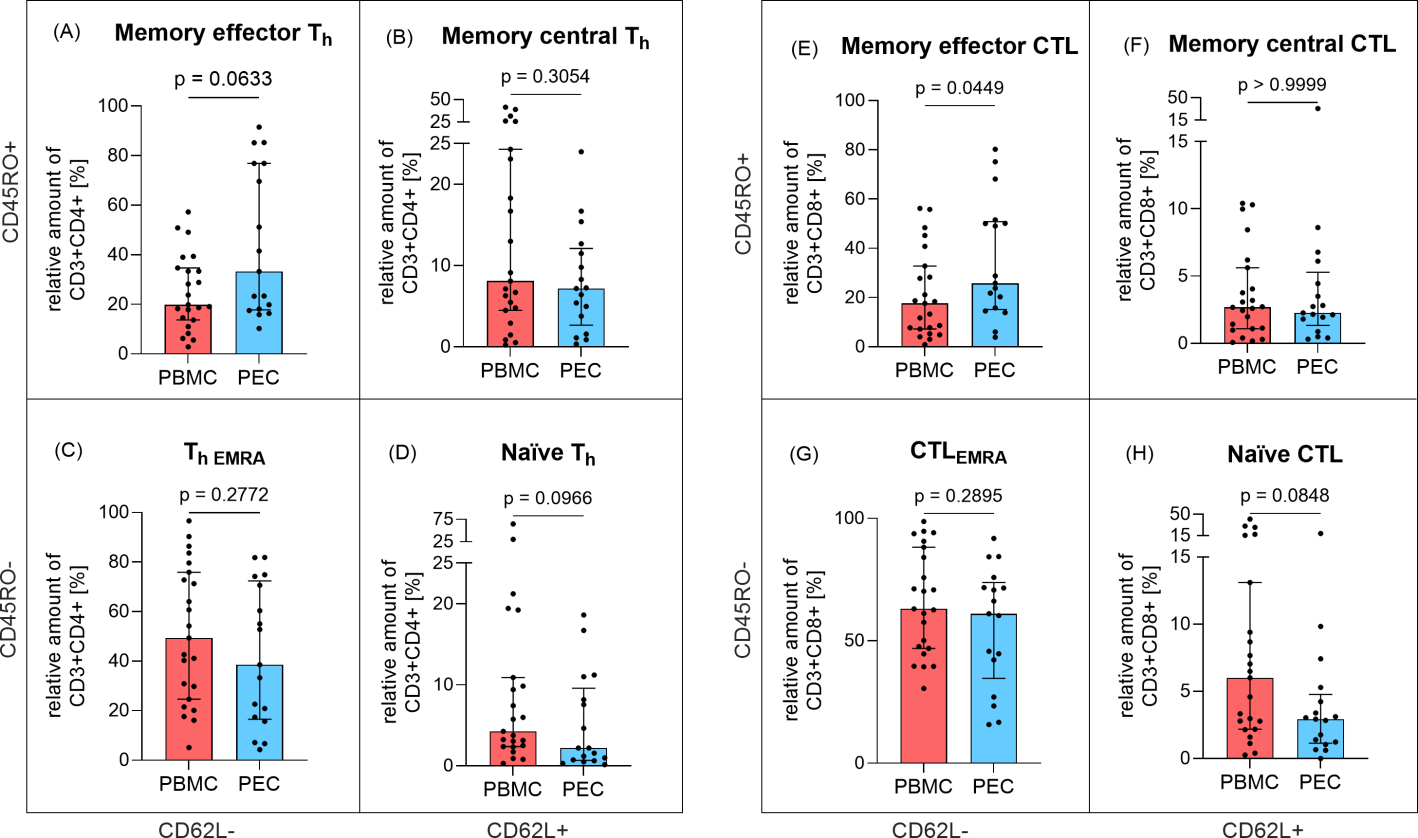
Expression of memory T cells in peripheral blood and pleural effusion. The proportion of T_h_ cell (CD3^+^CD4^+^) subpopulations **(A)** memory effector (CD45RO^+^CD62L^-^), **(B)** memory central (CD45RO^+^CD62L^+^), **(C)** effector memory re-expressing CD45RA, EMRA (CD45RO^-^CD62L^-^) and **(D)** naïve (CD45RO^-^CD62L^+^) as well as CTL (CD3^+^CD8^+^) subpopulations **(E)** memory effector (CD45RO^+^CD62L^-^), **(F)** memory central CTL (CD45RO^+^CD62L^+^), **(G)** effector memory re-expressing CD45RA, EMRA CTL (CD45RO^-^CD62L^-^), **(H)** naïve CTL (CD45RO^-^CD62L^+^) in relation to parent cell population was determined by flow cytometry in PBMC (red, n=23) and PEC (blue, n=17). Bars represent median value, error bars represent interquartile range, dots are drawn as individual data points. Group differences were assessed using two-tailed Mann-Whitney-U-test.

With respect to the expression of maturation markers CD45RO and CD62L, we also analyzed four T_h_ (**Figures 2A–D**) and CTL (**Figures 2E–H**) subpopulations each. The levels of memory central T_h_ cells (CD3^+^CD4^+^CD45RO^+^CD62L^+^, **Figure 2B**) and effector memory re-expressing CD45RA T cells (T_hEMRA_, CD3^+^CD4^+^CD45RO^-^CD62L^-^, **Figure 2C**) were comparable between PBMC and PEC. A similar finding emerged regarding the differences between PBMC and PEC for memory central CTL (CD3^+^CD8^+^CD45RO^+^CD62L^+^, **Figure 2F**) and effector memory re-expressing CD45RA T cells (CTL_EMRA_, CD3^+^CD8^+^CD45RO^-^CD62L^-^, **Figure 2G**). In contrast, memory effector CTL (CD3^+^CD8^+^CD45RO^+^CD62L^-^) were more abundant in PEC compared to PBMC (p=0.0449, **Figure 2A**) and memory effector T_h_ cells (CD3^+^CD4^+^CD45RO^+^ CD62L^-^) were slightly increased in PEC (p=0.0633, **Figure 2E**). Numbers of naïve T_h_ cells (CD3^+^CD4^+^CD45RO^-^CD62L^+^, p=0.0966, **Figure 2D**) and naïve CTL (CD3^+^CD8^+^CD45RO^-^CD62L^+^, p=0.0848, **Figure 2H**), where similar in PEC compared to PBMC.

#### 3.1.2 Lower NK and B cell frequency is associated with increased monocyte frequency in PEC

The sample volume was high enough for the analysis of thirteen B cell subpopulations, three natural killer (NK) cell subpopulations, and three monocyte subpopulations, respectively, in four PBMC and seven PEC samples (**Table 2**). Apart from innate B cells (CD19^+^CD27^-^IgD^-^IgM^-^), all investigated B cell populations tend to be quantitatively reduced in PEC compared to PBMC. In contrast, monocytes and their subpopulations showed a tendency towards stronger expression in the PEC samples, with the exception of non-classical monocytes (CD14^dim^CD16^+^), which showed comparable expression in both PBMC and PEC. However, the proportion of NK cell subsets was nearly equal in both, PBMC and PEC.

**Table 2:** Descriptive statistics of flow cytometry data: B cells, monocytes and NK cells.

Leukocyte subpopulation	Definition/cell surface markers	Sample	n	Median [%]	min - max	*p*-value
	*(Quantification in relation to)*					
B cells	CD19^+^	PBMC	4	17.0	7.7 – 25.8	0.11
	*(viable cells)*	PEC	7	7.2	1.6 – 28.2	
innate B cells	CD19^+^CD27^-^IgD^-^IgM^-^	PBMC	4	4.5	1.9 – 5.6	0.23
	*(CD19^+^)*	PEC	7	8.9	1.8 – 83.9	
naïve B cells	CD19^+^CD27^-^IgD^+^IgM^+^	PBMC	4	40.7	29.4 – 53.4	0.53
	*(CD19^+^)*	PEC	7	13.5	0.8 – 57.1	
memory B1 cells	CD19^+^CD27^+^IgD^-^	PBMC	4	11.7	1.4 – 17.7	0.65
	*(CD19^+^)*	PEC	7	3.7	2.7 – 29.1	
memory B2 cells	CD19^+^CD27^+^IgD^+^	PBMC	4	25.8	2.7 – 30.7	0.53
	*(CD19^+^)*	PEC	7	5.2	0.4 – 39.4	
marginal zone memory B cells	CD19^+^CD27^+^IgD^+^IgM^+^	PBMC	4	25.6	2.7 – 29.5	0.53
	*(CD19^+^)*	PEC	7	5.1	0.4 – 39.2	
IgM memory B cells	CD19^+^CD27^+^IgD^-^IgM^+^	PBMC	4	7.2	1.0 – 12.7	0.53
	*(CD19^+^)*	PEC	7	2.2	1.3 – 27.2	
class-switched memory B cells	CD19^+^CD27^+^IgD^-^IgM^-^	PBMC	4	3.2	0.4 – 7.8	0.93
	*(CD19^+^)*	PEC	7	1.7	0.6 – 3.3	
late memory B cells	CD19^+^CD27^+^CD38^+^IgM^+^	PBMC	4	13.0	1.3 – 14.8	0.53
	*(CD19^+^)*	PEC	7	1.4	0.5 – 45.5	
plasmablasts	CD19^+^CD27^+^CD38^+^IgM^-^	PBMC	4	1.0	0.1 – 2.8	0.53
	*(CD19^+^)*	PEC	7	0.5	0.2 – 1.1	
transitional B cells	CD19^+^CD20^+^CD27^-^CD38^+^	PBMC	4	10.5	7.8 – 24.7	0.53
	*(CD19^+^)*	PEC	7	8.7	0.3 – 21.2	
B1 cells	CD20^+^CD27^+^CD43^+^	PBMC	4	11.1	3.4 – 15.7	0.99
	*(CD20^+^)*	PEC	7	9.8	1.5 – 35.9	
B2 cells	CD20^+^CD27^+^CD43^-^	PBMC	4	23.2	1.3 – 31.9	0.41
	*(CD20^+^)*	PEC	7	11.6	0.4 – 28.0	
monocytes	CD14^+^	PBMC	4	12.5	0.9 – 16.3	0.53
	*(viable cells)*	PEC	7	16.8	0.9 – 79.0	
classical monocytes	CD14^+^CD16^-^	PBMC	4	9.1	0.6 – 17.7	0.65
	*(viable cells)*	PEC	7	10.0	0.5 – 34.1	
intermediate monocytes	CD14^+^CD16^+^	PBMC	4	3.1	0.4 – 4.7	0.41
	*(viable cells)*	PEC	7	6.8	0.3 – 44.8	
non-classical monocytes	CD14^dim^CD16^+^	PBMC	4	1.0	0.8 – 2.4	0.29
	*(viable cells)*	PEC	7	0.7	0.0 – 6.6	
Total natural killer (NK) cells	CD3^-^CD14^-^CD19^-^CD20^-^CD16^+^CD56^bright^, CD3^-^CD14^-^CD19^-^CD20^-^CD16^+^CD56^dim^ and CD3^-^CD14^-^CD19^-^CD20^-^ CD16^-^CD56^bright^	PBMC	4	8.9	6.9 – 45.1	0.93
	*(viable cells)*	PEC	7	12.2	2.3 – 19.7	
CD16^+^CD56^bright^ NK cells	CD3^-^CD14^-^CD19^-^CD20^-^CD16^+^CD56^bright^	PBMC	4	9.0	2.8 – 26.1	0.65
	*(total NK cells)*	PEC	7	13.1	2.4 – 44.2	
CD16^+^CD56^dim^ NK cells	CD3^-^CD14^-^CD19^-^CD20^-^CD16^+^CD56^dim^	PBMC	4	60.3	9.2 – 92.4	0.79
	*(total NK cells)*	PEC	7	54.6	5.8 – 86.4	
CD16^-^CD56^bright^ NK cells	CD3^-^CD14^-^CD19^-^CD20^-^ CD16^-^CD56^bright^	PBMC	4	21.2	4.8 – 82.5	0.93
	*(total NK cells)*	PEC	7	26.0	11.2 – 83.6	

The proportion of B cells, monocytes, NK cells and their respective subpopulations in relation to parent cell population was determined by flow cytometry in PBMC (n=4) and PEC (blue=7). Data is given as median as well as minimum and maximum values (min – max). Group differences were assessed using two-tailed Mann-Whitney-U-test.

### 3.2 Total protein analysis

**Figure 3:**
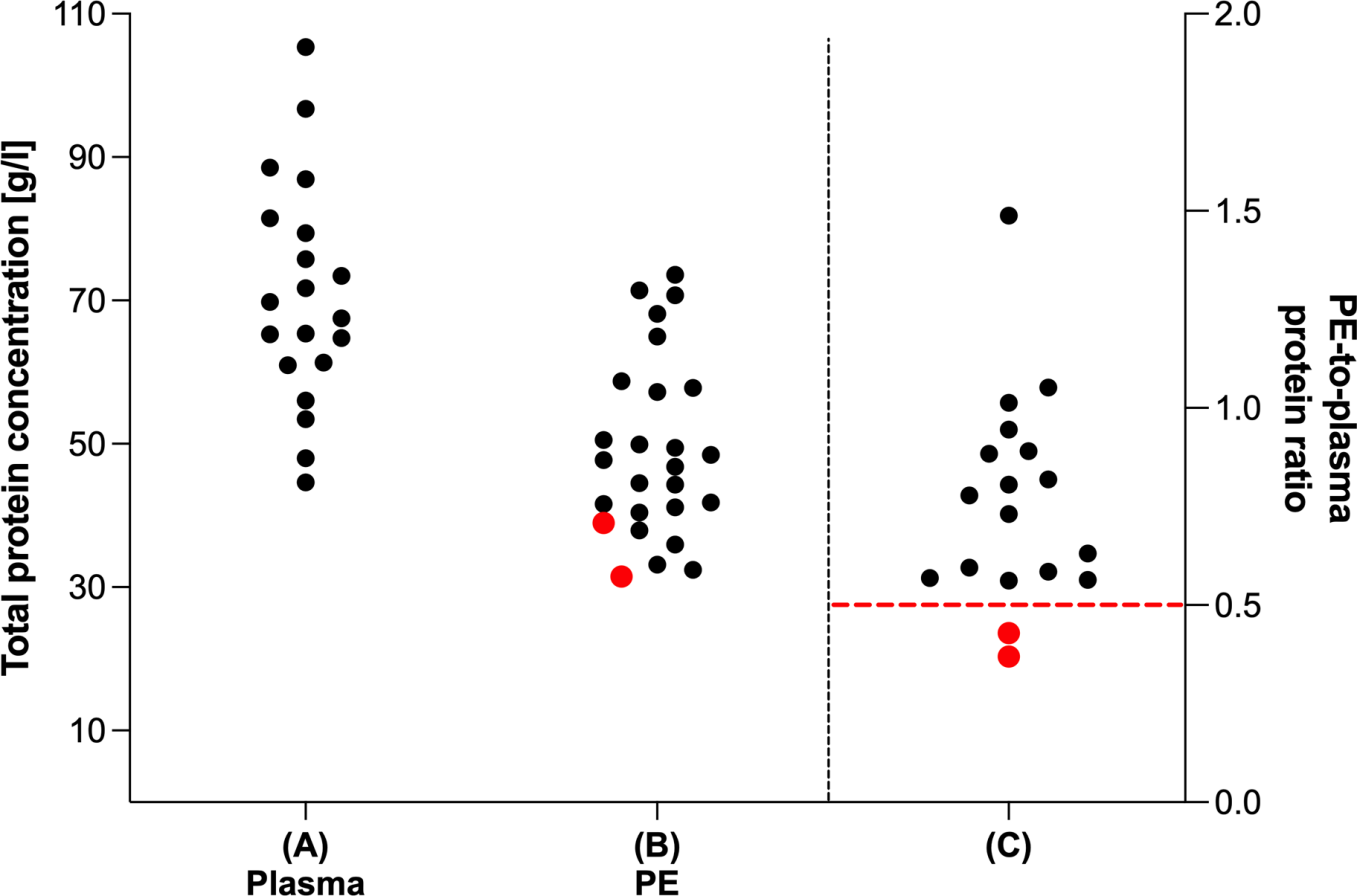
Total protein analysis of plasma and pleural effusion. Total protein level (g/l) of **(A)** plasma (n=20) and **(B)** cell free fraction of PE samples (n=26). **(C)** Ratio of total protein level of cell free fraction of PE samples and their corresponding plasma samples (n=18). Total protein analysis was performed using Pierce™ BCA Protein Assay. Red dots indicate PE samples categorized according to Light’s criteria as transudates as red dashed line indicates limit value for categorization from transudates to exudates (PE-to-plasma protein ratio < 0.5).

Total protein concentration was analyzed in both, plasma and PE samples (**Figure 3**). While median total protein concentration of plasma was 68.65 g/l (IQR: 61.05–80.95, range: 44.59–105.4, **Figure 3A**), the median total protein concentration of cell free supernatant of PE was 47.28 g/l (IQR: 40.06–58.07, range: 31.51–73.59, **Figure 3B**). The PE-to-plasma protein ratio was determined in 18 patients. Notably, in 16 of 18 analyzed patients the ratio exceeded 0.5 (**Figure 3C**). Thus, according to Light’s criteria, 89% of the analyzed PE were categorized as exudates.

### 3.3 Different cytokine levels in plasma and pleural effusion

**Figure 4:**
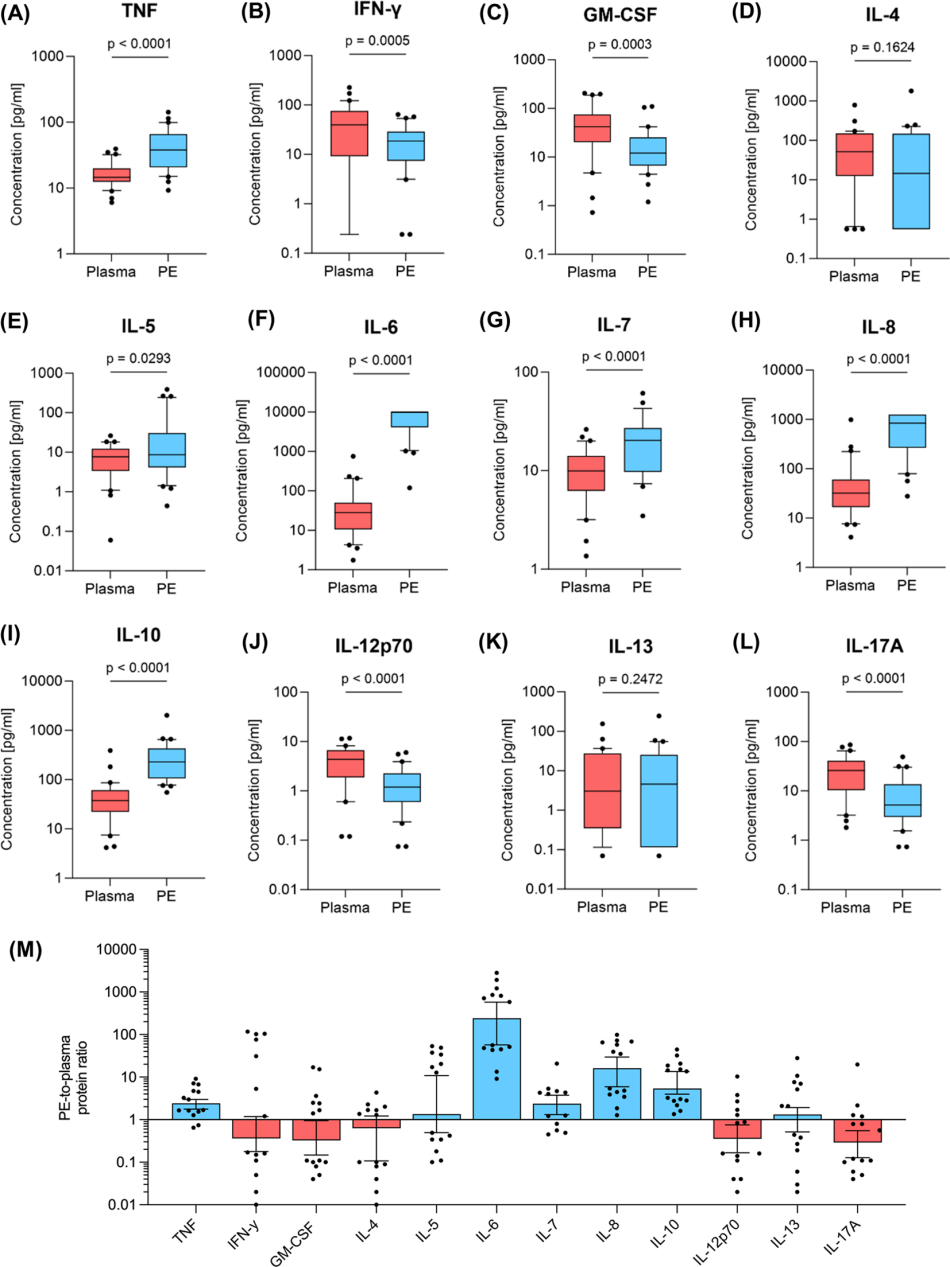
Cytokine levels in plasma and pleural effusion. The amount of **(A)** TNF, **(B)** IFN-γ **(C)** GM-CSF, **(D)** IL-4, **(E)** IL-5, **(F)** IL-6, **(G)** IL-7, **(H)** IL-8, **(I)** IL-10, **(J)** IL-12p70, **(K)** IL-13, **(L)** IL-17A was determined by multiplex immunoassay in pleural effusion (PE, blue, n=30) and peripheral blood samples (red, n=30). Whiskers extend from the 10^th^ to the 90^th^ percentiles. Points below and above the whiskers are drawn as individual data points. Specific protein ratios of pleural effusion and corresponding samples of plasma are given in **(M)**. Group differences were assessed using two-tailed Wilcoxon matched-paired signed rank test.

A multiplex immunoassay was employed to quantify twelve cytokines in paired samples of plasma and in the cell-free supernatant of the corresponding PE samples (**Figure 4**).

Comparative analysis revealed significantly higher cytokine levels in PE compared to plasma for the inflammation-associated cytokines TNF (**Figure 4A**) and IL-6 (**Figure 4F**), as well as IL-7 (**Figure 4G**) and IL-8 (**Figure 4H**) (p<0.0001, respectively). Additionally, T_h_2 associated cytokines IL-4 (**Figure 4D**) and IL-13 (**Figure 4K**) showed no significant differences between PE and plasma, whereas IL-5 (**Figure 4E**) and IL-10 (**Figure 4I**), which are also produced by T_h_2, exhibited significant higher cytokine levels in PE. Conversely, T_h_1-associated IL-12p70 (**Figure 4J**) and IFN-γ (**Figure 4B**) were lower in PE, as well as GM-CSF (**Figure 4C**) and IL-17A (**Figure 4L**) (p ≤ 0.0005).

PE-to-plasma protein ratio were also determined for cytokines using adapted Light’s criteria (**Figure 4M**). The concentrations of five of the quantified cytokines were lower in PE compared to plasma: GM-CSF, IFN-γ, IL-4, IL-12p70 and IL-17A. T_h_1-associated IL-12p70 and IFN-γ showed PE-to-plasma protein ratios of 0.36. GM-CSF, IL-4 and IL-17A were between 1.6- and 3.4-fold lower in PE compared to plasma. Conversely, the TNF, IL-5, IL-7, IL-10 and IL-13 concentrations were up to 10-fold higher in PE than in plasma, with IL-8 showing more than a 10-fold increase and IL-6 exceeding a 242-fold increase in PE compared to plasma.

### 3.4 Clinical factors influence cytokine levels in plasma and pleural effusion

**Figure 5:**
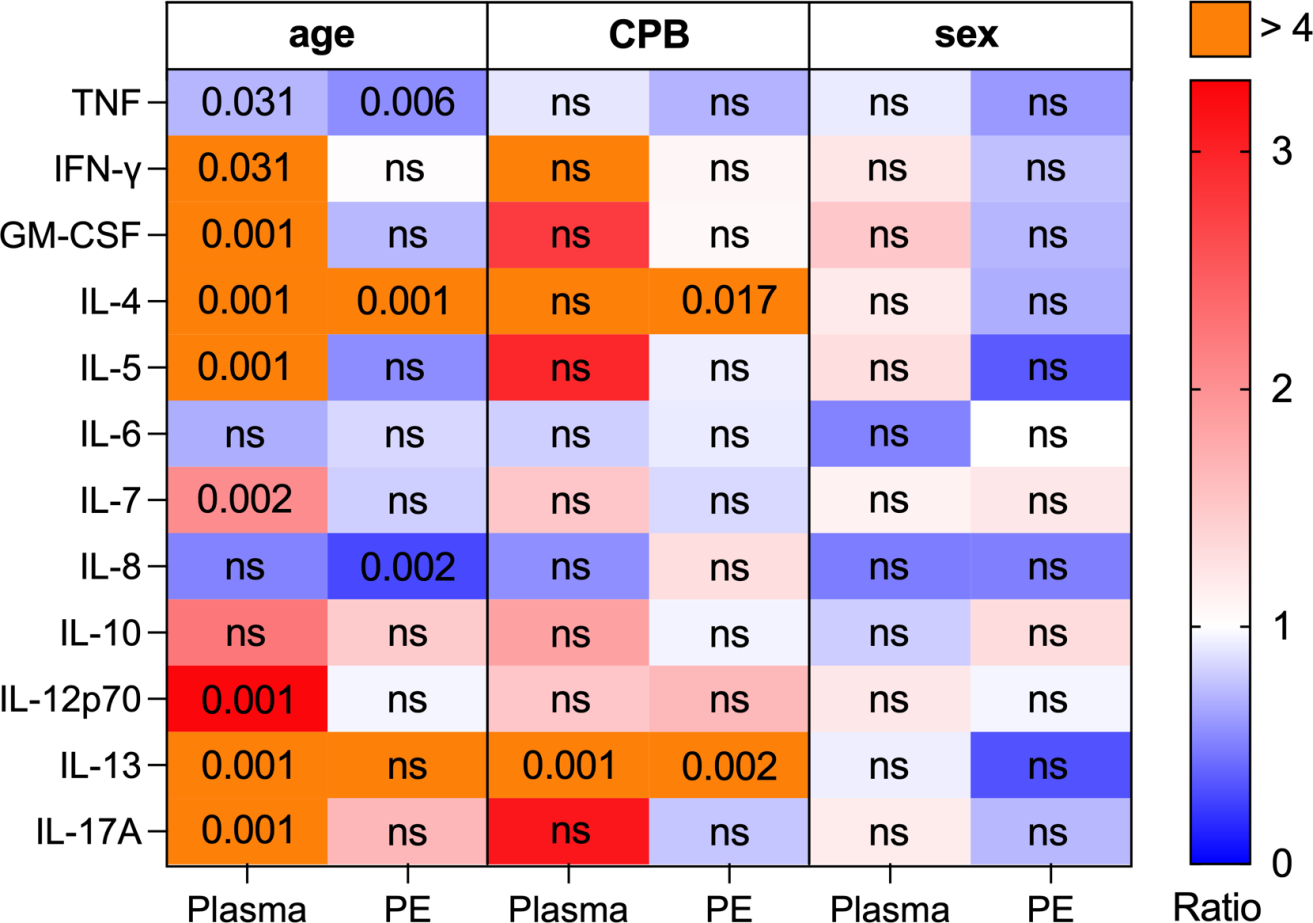
Cytokine ratios in plasma and pleural effusion regarding clinical factors (age, CPB & sex). The amount of TNF, IFN-γ, GM-CSF, IL-4, IL-5, IL-6, IL-7, IL-8, IL-10, IL-12p70, IL-13, IL-17A was determined by multiplex immunoassay in plasma (n=30) and PE samples (n=30). Patients were grouped according to their age (<12 months, n=13; >12 months, n=17), using of a CPB during surgery (CPB, n=16; no CPB, n=14) and patient’s sex (female, n=9, male, n=21). For each cytokine the following three ratios were assessed in plasma and PE: (1) <12 months/>12 months, (2) CPB/no CPB, (3) male/female. Ratio levels are connected to the color code (blue: ratio < 1, white: ratio = 1, red: ratio > 1, purple: ratio > 4). The differences in the expression of the cytokines in either plasma or PE were assessed between the groups of age, CPB and sex using Mann-Whitney-U test. Not significant (ns) and significant p-values (individual values; p < 0.05) are shown in corresponding cells of the matrix.

The influence of clinical factors on cytokine levels in both plasma and PE was analyzed (**Figure 5**). Factors assessed included gender (male vs. female), age (<12 months of age vs. >12 months) and use of cardiopulmonary bypass (CPB) (CPB vs. no CPB). The use of CPB resulted in significantly higher levels of IL-4 and IL-13 in PE compared to cardiac surgery without CPB (p=0.02 and p=0.01, respectively). Additionally, plasma levels of IL-13 were significantly elevated in patients with CPB (p=0.01). Comparisons between age groups revealed significantly higher plasma cytokine levels in patients aged <12 months for IL-4, IL-5, IL-7, IL-12p70, IL-13, IFN-γ and GM-CSF. However, in PE from patients <12 months only IL-4 was elevated (p=0.001). Patients >12 months exhibited significantly increased levels of IL-8 (p=0.016) in PE, while corresponding plasma samples did not differ in IL-8 levels between the two age groups. TNF levels in both PE and plasma samples showed significant increases in patients >12 months (p=0.006 and p=0.031, respectively). No significant differences in cytokine levels were observed between genders in either plasma or PE. Descriptive statistics for multiplex immunoassay data are provided in **Supplementary File 5** in **Supplementary Data Sheet 1** (age), 6 (CPB) and 7 (sex).

### 3.5 Correlation between immune cells and cytokines in blood and pleural effusion

**Figure 6:**
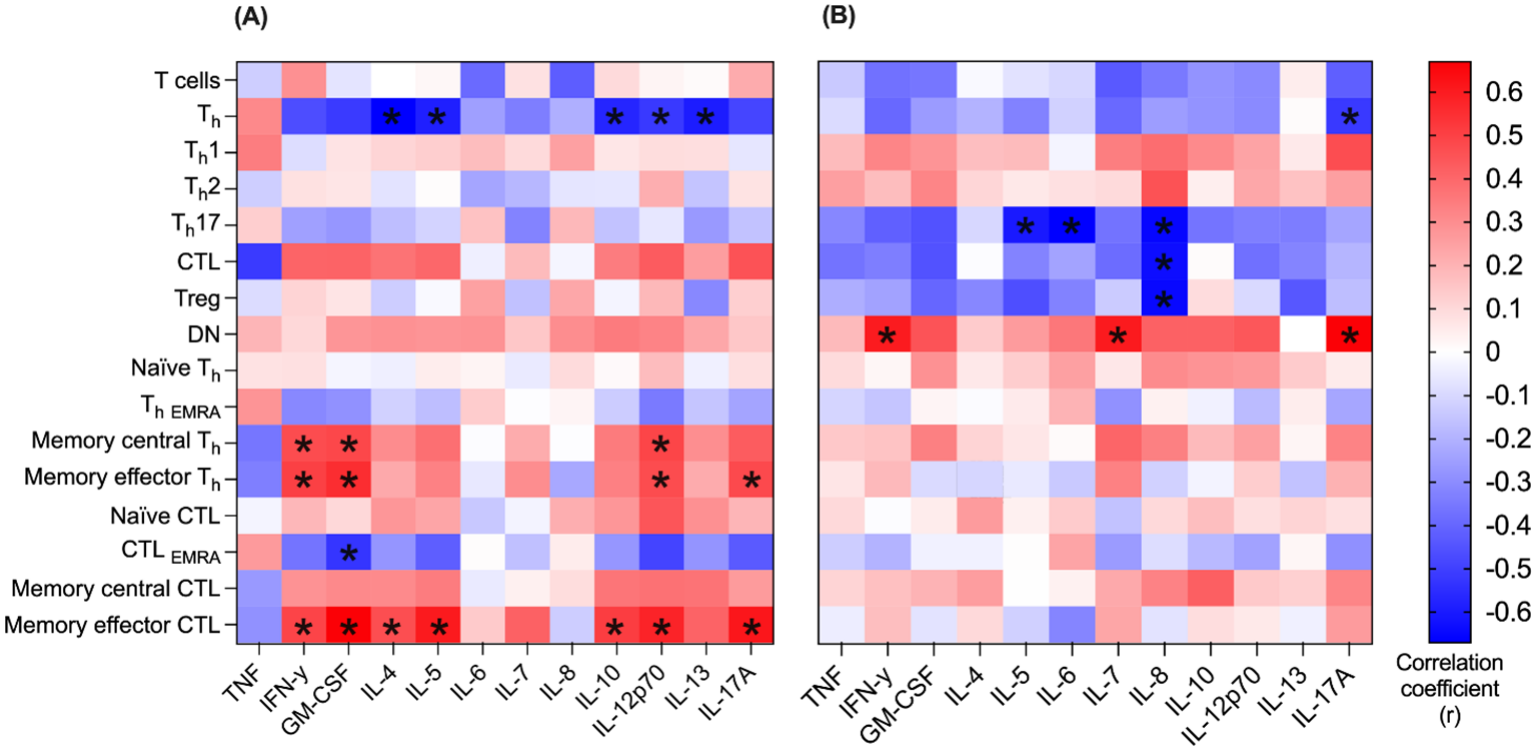
Correlation between immune cells and cytokines in blood and pleural effusion. Correlation between n=16 T cell populations and n=12 cytokines was determined in both blood **(A)** and pleural effusion **(B)**. Spearman’s rank correlation was used to determine the correlation coefficient (r) of every combination of T cell population and cytokine. Correlation coefficient (r) is represented by the color code (blue: r = -0.6, white: r = 0, red r = +0.6). For each correlation coefficient (r), the corresponding p-value is given. Only significant p-values are marked below (*p < 0.05).

Correlation between 16 T cell populations and 12 cytokines was determined in both blood and pleural effusion (**Figure 6**). In blood, especially memory T cells correlated significantly positively with multiple cytokines. Both memory effector (CD3^+^CD4^+^CD45RO^+^ CD62L^-^) and memory central T_h_ cells (CD3^+^CD4^+^CD45RO^+^ CD62L^+^) showed positive correlation for IL-12p70, IFN-γ and GM-CSF (r = 0.4, r = 0.5, r = 0.4 and r = 0.4, r = 0.4, r = 0.4; and p<0.05 for each). Furthermore, memory effector T_h_ cells correlated positively with IL-17A in blood (r = 0.4, p<0.05). Memory effector CTL correlated significantly positively with seven different cytokines in blood (**Figure 6A**), inter alia, GM-CSF, IFN- γ and IL-12p70 (r = 0.6, r = 0.4 and r = 0.5, respectively and p<0.05 for each). In pleural effusion the only cell population with significant positive correlation coefficient for any of the specific cytokines was DN T cells (CD3^+^CD4^-^CD8^-^). They correlated positively with the levels of IL-7, IL17A and IFN-γ (r = 0.6, r = 0.6 and r = 0.7, respectively and p<0.05 for each). Significant negative correlations were found between six T cell populations and corresponding cytokines, too. A particularly notable example is T_h_17 cells, which correlated strongly negatively with the levels of IL-5, IL-6 and IL-8 in pleural effusion (r = -0.6, r = -0.7 and r = -0.7, respectively and p<0.05 for each).

## 4 Discussion

In this study, we performed a comparative characterization of the immune response at the cellular and cytokine level in both peripheral blood and pleural effusion (PE) samples from pediatric patients following cardiac surgery. Previous characterizations of PE have largely been retrospective and focused on biochemical parameters or a limited analysis of immune cells in small patient cohorts. The variability of methods used in these studies makes direct comparisons difficult. Thus, hitherto, the immune compartment of the pleural cavity was poorly understood. We simultaneously analyzed multiple lymphocyte subpopulations and their associated cytokines in blood and PE of pediatric cardiopathic patients using validated methods. The selection of cells and cytokines is based on previous studies and has been expanded as far as possible (32, 33). The aim of the study was to provide the first comprehensive immunological insight into corresponding blood and PE samples in order to achieve a more detailed understanding of the immunological processes involved in the pathogenesis of PE after pediatric cardiac surgery that might have an impact on the prediction and treatment.

Improving the management of PE after pediatric cardiac surgery is urgently needed, as PE is a particularly common complication in children after cardiac surgery: The reported incidence in children is up to 39%, compared with only around 7% in adults after cardiac surgery (4). PE leads to longer ICU and hospital stays, a higher morbidity and potentially to increased susceptibility to infections (41). The increased susceptibility to infection has primarily been discussed in the context of chylothorax, which is a postoperative complication after corrective surgery of congenital heart defects, which mainly occurs in neonates (42) and leads to the loss of large amounts of fats, vitamins, antibodies (33, 43) and lymphocytes (10, 11, 32, 33).

PE after cardiac surgery can be either transudates or exudates. Transudates result from altered hydrostatic or oncotic pressures with intact pleural structures, while exudates arise from inflammation and subsequent damage to pleural structures, particularly endothelial and mesothelial cells (44, 45). Since the presence of any Light’s criteria is sufficient to diagnose an exudate (39, 46), we focused on the PE-to-plasma protein ratio and classified all but one PE sample as exudates. The presence of immunological components in PE is characteristic of exudates (47–49), as only damaged pleural structures permit transition of leukocytes and cytokines from blood to the pleural cavity (45, 50).

The transition of leukocytes from the peripheral blood into the pleural effusion with subsequent puncture and drainage removes leukocytes from the systemic circulation. Previous studies show a reduction in the absolute number of T cells, T_h_, CTL and B cells in the peripheral blood below age-related normal values (32, 33). Orange et al. showed an increase in the relative number of T cells in PE when comparing leukocyte populations in blood and PE, while the opposite was observed for NK cells (33). In contrast, the results of our study are more in line with those of Prelog et al. who found no significant difference between blood and PE for major populations such as T, B and NK cells (32). However, by analyzing a wide range of smaller subpopulations, we were able to detect differences in the relative amounts of leukocyte subpopulations between blood and PE, which may help to better understand the mechanisms underlying cellular immune response in the presence of PE.

Specifically, we observed a slightly reduction in the number of T cells in PE, with T_h_ cells and CTL present in nearly equal proportions in both compartments. These results are consistent with previous observations (32, 33). Furthermore, we expanded these findings by analyzing a broader range of T cell subpopulations and their corresponding cytokine levels to better understand the mechanisms underlying local and systemic T cell immune responses.

T_h_ cells are typically the dominant T cell population in blood and outnumber CTL (51, 52). The same type of distribution could be demonstrated in PE by calculating a T_h_/CTL ratio, which was skewed towards T_h_ cells. For a more profound understanding of the T_h_ cell immune response, we analyzed T_h_1, T_h_2 and T_h_17 cells as well as their corresponding cytokines. Evolutionarily, T_h_1 immune responses are optimized to combat acute infections. A meta-analysis by Zeng et al. showed that T_h_1 immune responses dominates in tuberculous PE (53). In our study investigating cardiopathic PE, T_h_1 cells were significantly increased in PE compared to PBMC, whereas T_h_2 cells were present in nearly equal proportions in both compartments. The T_h_1/T_h_2 ratio was skewed towards T_h_1 dominance in PE, primarily due to elevated T_h_1 numbers. Interestingly, the levels of key T_h_1 cytokines IL-12p70 and IFN-γ were lower in PE compared to plasma, while TNF levels were significantly lower in plasma, possibly contributing to the enhanced T_h_1 cell response in PE.

Overall, there was no quantitative difference in the levels of the T_h_2 cells or the anti-inflammatory, T_h_2-associated cytokines IL-4 and IL-13 between the blood and the PE samples. This suggests that the T_h_2 immune response is similar in both compartments. Notably, the levels of both IL-5 and IL-10, which are also anti-inflammatory cytokine produced by T_h_2 cells, were significantly elevated in PE.

Alongside T_h_2 cells, T_regs_ are the main producers of IL-10 (54, 55). However, the proportion of T_regs_ was nearly identical in both, PBMC and PE and do not correspond to the significantly higher IL-10 levels in PE. This suggests that while T_regs_ play a limited role in this context, the elevated IL-10 levels could be attributed to production by other immune cells, such as T_h_1, T_h_2, T_h_17 and macrophages (56). IL-10 might suppress the expression of pro-inflammatory cytokines in PE. The level of IL17A and GM-CSF, both of which can be secreted by T_h_17 cells (56), were significantly lower in PE compared to plasma, indicating that T_h_17 activity might not be critical for pathogen defense in this setting, although the number of T_h_17 cells in PE is significantly elevated in comparison to plasma. This hypothesis might be supported by the markedly high levels of IL-6 in the PE, which might suggest an acute inflammatory response. To explain this, IL-6 is mainly produced in the initial stage of inflammation and thus stimulates the synthesis of acute phase proteins such as CRP and serum amyloid A. In turn, TNF continues to stimulate the synthesis of IL-6. Appropriately, both TNF and IL-6 were significantly elevated in PE in the present study. However, it is important to note that IL-6 acts through two different signaling pathways, only one of which is specifically upregulated during inflammation (57, 58).

Double-negative T cells (DN T cells, CD3^+^CD4^-^CD8^-^) are involved in both pro- and anti-inflammatory immune responses and present approximately 3-5% of T cells in blood (59, 60). Interestingly, in our study DN T cells were up to 10% in blood and the values in PE were even almost twice as high as in blood. This elevated level of DN T cells in PE may be due to their high potential of migrating from blood to peripheral tissues (61). There are different subtypes of DN T cells, including cytotoxic and T_h_-like DN. The latter are capable of producing cytokines like IL-4, IL-17 and IFN-γ (60). In fact, our findings revealed a significant positive correlation between DN T cells and the cytokines IL-17A and IFN-γ in pleural effusions, indicating the presence of T_h_-like DN in PE. In contrast to CTL and T_h_ cells, which mainly express αβ-positive T cell receptors, DN T cells express either αβ-positive T cell receptor (TCRαβ^+^) or γδ-positive T cell receptor (TCRγδ ^+^) (60). TCRγδ^+^ cells are of particular interest in pleural effusions immune response as they are involved in chemokine and cytokine production and wound healing processes (62, 63). While we infer the presence of TCRγδ cells due to their strong representation in DN T cells, specific flow cytometric detection of TCRγδ cells is recommended in future studies.

T cell development, maintenance and restoration of mature T cell homeostasis is strongly dependent on IL-7. IL-7, predominantly produced by non-hematopoietic stromal cells and dendritic cells, promotes the transition from naïve (CD45RO^-^) to memory (CD45RO^+^) T_h_ cells. Significantly increased IL-7 levels in PE may reflect a compensatory response to lymphopenia through enhanced T cell proliferation (64). IL-7 also plays a critical role in B cell lineage commitment and Ig gene rearrangement during B cell development (65–68). Thus, further detailed analyses of B cells in PEC could be valuable. IL-7 is also crucial for the development and maintenance of many members of the ILC (innate lymphoid cell) family (69, 70), contributing to the formation of lymphoid structures and barrier defense mechanisms (64). Thus, IL-7 may be a key cytokine in the context of PE. Additionally, the observed increase in CD45RO expression indicates that naïve T_h_ cells and CTL were exposed to antigens, leading to immunological activation (71). This phenomenon has been primarily observed in malignant pleural effusions (24, 26, 72), but in the present context, activation may be driven by the congenital heart disease itself or by the cardiac surgery.

Our results suggest that both local and systemic immunological factors, as well as their mutual influence, are involved in the immune response in PE after pediatric cardiac surgery. Cardiac surgery leads to systemic changes in the immune system, which can favor the development of PE. This is due to changes in endothelial permeability status through enhanced T_h_2 response (73) and higher expression of adhesion molecules like ICAM and L-selectin (CD62L) (74), which are involved in both endothelial transmigration of leukocytes and inflow of cytokines into the pleural cavity.

In addition to systemic inflammation, pleural structures, particularly mesothelial cells, contribute to a local immune response. Mesothelial cells exhibit phagocytic activity (75, 76), antigen present capabilities (77, 78), and produce chemokines and cytokines (77–79). Fujino et al. showed that mesothelial cells are highly capable of producing IL-6 (79) which may contribute to the extremely elevated levels of IL-6 in PE compared to blood found in the present study. Local production of cytokines could also occur from leukocytes that have migrated into the pleural cavity. Prelog et al. described an increased expression of activation markers on pleural effusion cells, which may contribute to local cytokine milieu (32). Consequently, cytokines in the PE can originate from both systemic and local sources, while PE cells are likely of systemic origin, given the lack of local cells production.

However, transmigration of cells into the pleural cavity is influenced by local chemokines, such as IL-8. IL-8 is secreted by various cell types, including monocytes, neutrophils, basophils, epithelial, endothelial and mesothelial cells in response to inflammatory stimuli. It recruits leukocytes to sites of local inflammation and plays a crucial role in wound healing by promoting fibroblast migration (80, 81). We found strongly elevated IL-8 levels in PE, which represents a concentration gradient towards the pleural cavity. Inflammatory stimuli that induce IL-8 secretion include lipopolysaccharide and TNF (59), the latter of which we also found to be locally elevated in PE. TNF, a pro-inflammatory cytokine, is primarily produced by activated macrophages and T lymphocytes, as well as NK cells.

In addition to chemokine attraction, surface markers on leukocytes are necessary for cellular migration. The surface marker CD62L may play a role in navigating T cells between the blood and PE. While Prelog et al. hypothesized that the homing factor CD62L is involved in the migration of cells from blood capillaries into PE (32), our data does not support this, as CD62L-negative T_h_ cells and CTL outnumber their CD62L-positive counterparts in PE. In particular, memory effector T_h_ cells, characterized by CD45RO expression in the absence of CD62L, accumulated in PE, while naïve T cells accumulated among PBMC. This pattern was similarly observed in CTL subpopulations, suggesting that the different expression of CD45RO and CD62L is not unique to either T cell group, but may reflect a more general regulatory mechanism.

In addition to congenital heart disease and surgery itself, preoperative conditions can lead to elevated cytokine levels (73), complement factors (74), and altered expression of surface markers on monocytes and granulocytes in blood (82). Therefore, this study investigated the influence of clinical characteristics on the immune response in PE. Differences in gender, age and use of surgical techniques, are known to influence the immune system. The use of cardiopulmonary bypass during surgery was associated with elevated levels of T_h_2 cytokines, including IL-4 and IL-13, in PE. An increased T_h_2 response, which promotes the development of PE following heart surgery, has previously only been observed in blood samples (73).

Although gender-specific differences have been described in some immune responses (83–85), no such differences were observed in this study. Children under one year of age had higher levels of a broad range of cytokines than older children. The elevated cytokines could not be attributed to a specific type of immune response, but may indicate a more severe overall immune response in younger children. Interestingly this age-related difference was mainly observed in blood and not in PE. One limitation of the study is that due to the small sample volumes, a complete analysis of the B cell, monocyte and NK cell subpopulations was only possible in subsets of the patients. The simultaneous analysis of other immune cells such as dendritic cells and possibly existing neutrophils as well as the implementation of a leukocyte marker would be beneficial in the future. The observatory nature of this study does not allow to strictly distinguish between systemic and local contributions to the observed inflammatory responses. For this purpose, a larger cohort of patients should be investigated to allow a comparative characterization of the immune response in traumatic and non-traumatic pleural effusions as well as chylothoraces. This could also address the unresolved question of whether mechanical or immunological traumatic factors, or both, are responsible for causing chylothorax. Additional gene expression analyses might help identifying the source of the cytokines. A better understanding of the immune modulators in PE and their activity following thoracic surgery could be beneficial for the design of targeted therapies and even preventing PE.

## Conclusion

The study offers new insights into immunological characteristics of pleural effusions and peripheral blood through the first combined comparative investigation of cytokines, total protein, and lymphocyte subpopulations in pediatric patients after cardiac surgery. The proportion of immune cells involved in immune modulation differs between peripheral blood and pleural effusion samples. Pro- and anti-inflammatory processes occur simultaneously in pleural effusions, with cytokine-level expression being more pronounced than differences at the cellular level. Traditionally, pleural effusions have been classified according to biochemical and microbiological parameters to guide appropriate therapy. A better understanding of the dynamic immunological processes within pleural effusions may improve the prediction and management of pleural effusion.

## Data Availability

The raw data supporting the conclusions of this article will be made available by the authors, without undue reservation.
