# Supplementary material for: Immunological characterization of pleural effusions in pediatric patients
Source: Front Immunol. 2024 Dec 16;15:1506073. doi: 10.3389/fimmu.2024.1506073 (PMC11682977; doi:10.3389/fimmu.2024.1506073)
Supplement: Supplementary file 1 [file DataSheet1.docx]

Supplementary Material

Immunological characterization of pleural effusions in pediatric patients

Luca Flögel^1^, Elisabeth Kaiser^1^, Muriel Charlotte Hans^1^, Sybelle Goedicke-Fritz^1^, Michelle Bous^1^, Hashim Abdul-Khaliq^2^, Martin Poryo^2^, Michael Zemlin^1^, Regine Weber^1^

^1^Department of General Pediatrics and Neonatology, Saarland University, Campus Homburg, Homburg/Saar, Germany

^2^Department of Pediatric Cardiology, Saarland University Medical Center, Homburg, Germany

*** Correspondence:** Regine Weber: [regine.weber@uks.eu](mailto:regine.weber@uks.eu)

# Supplementary Files


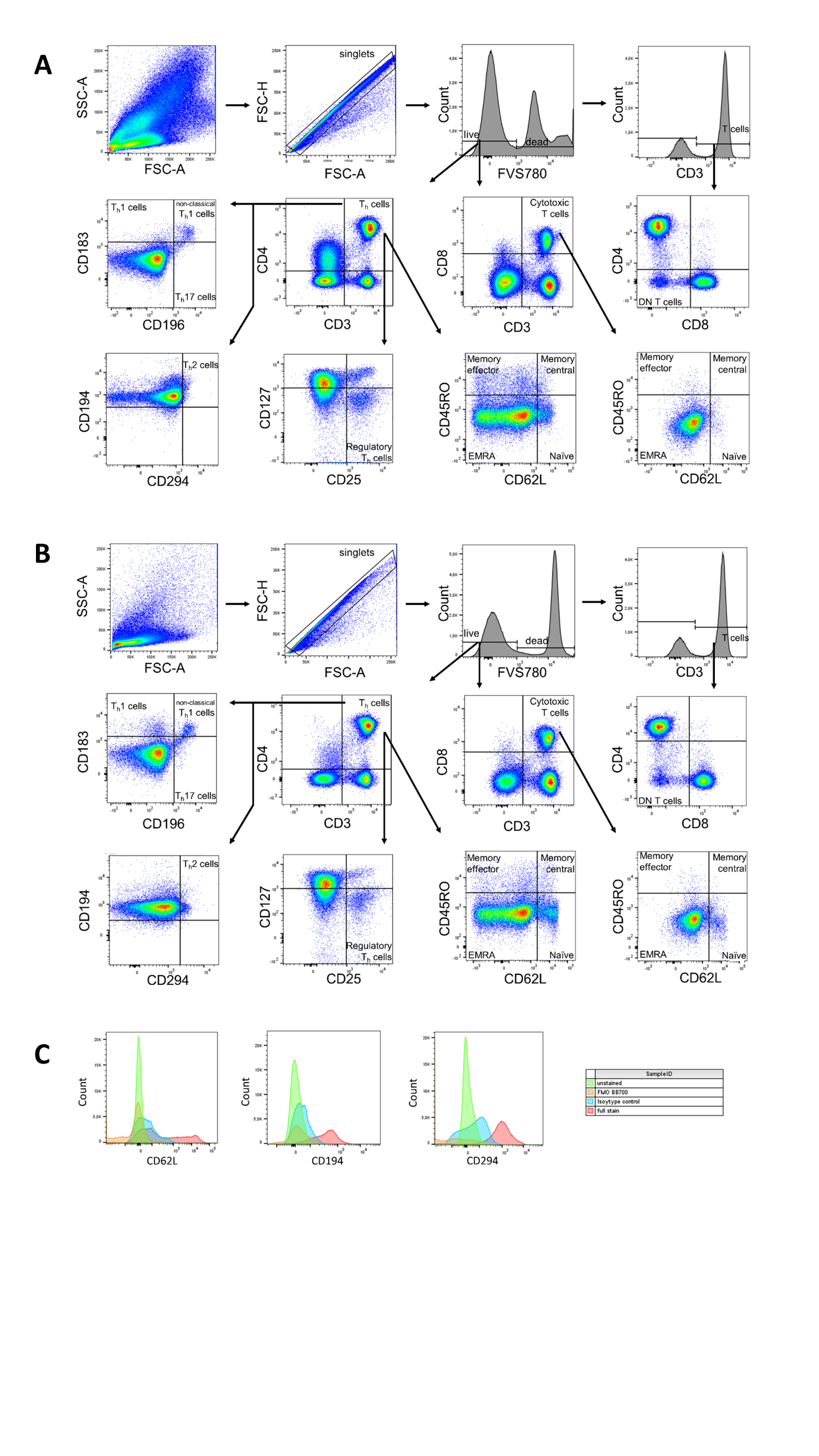


**Supplementary File 1:** Gating strategy (T cells)

Gating strategy of T cell subpopulations of a representative (A) PBMC and (B) PEC sample from a study participant. Analysis was performed using FlowJo (BD Biosciences). Gating was based first on morphological parameters (FSC-H/FSC-A) and FVS780 negative cells were considered as viable cells. Within the viable cells, the number of CD3^+^ cells was determined. The CD3^+^ fraction was divided into CD4^+^ T helper cells and CD8^+^ cytotoxic T cells and also double-negative (DN) CD4^-^CD8^-^ T cells are shown. Both CD4^+^ as well as CD8^+^ cell fractions were subdivided into naïve (CD45RO^−^CD62L^+^), EMRA (CD45RO^−^CD62L^−^), memory effector (CD45RO^+^CD62L^−^), and memory central (CD45RO^+^CD62L^+^). CD4^+^ T helper cells were identified as well as T_h_1 cells (CD183^+^CD196^-^), T_h_17 cells (CD183^-^CD196^+^) and T_h_2 cells (CD194^+^CD294^+^). Regulatory Th cells were identified as CD25^+^CD127^-^. (C): controls for positive staining of CD62L, CD194 and CD294 (color code: green: unstained, orange: FMO, blue: isotype control, red: full staining) using a representative PBMC sample.

**
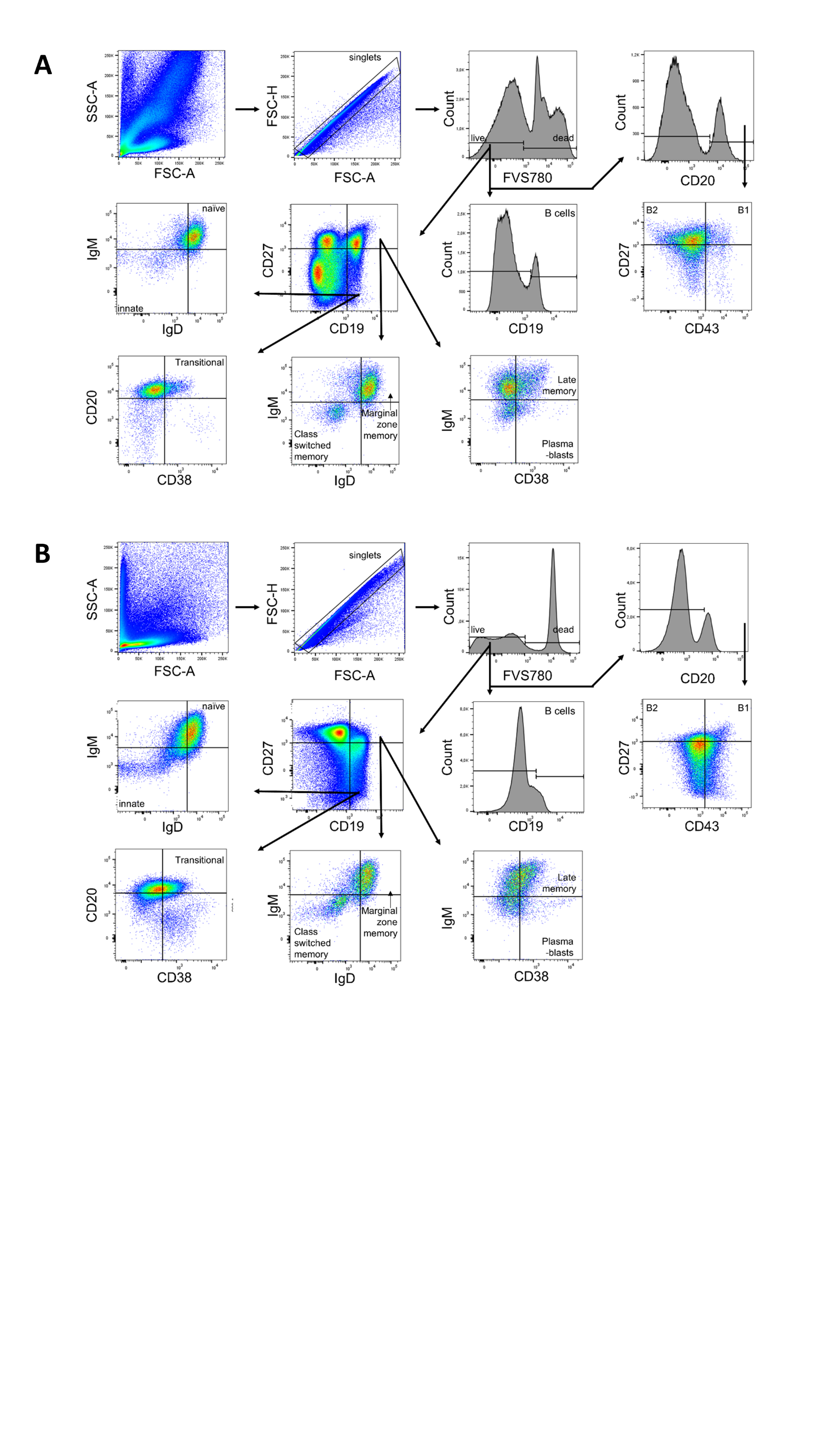
**

**Supplementary File 2:** Gating strategy (B cells)

Gating strategy of B cell subpopulations of a representative (A) PBMC and (B) PEC sample from a study participant. Analysis was performed using FlowJo (BD Biosciences). Gating was based first on morphological parameters (FSC-H/FSC-A) and FVS780 negative cells were considered as viable cells. Within the viable cells, the number of CD19^+^ and CD20^+^ cells was determined. The CD19^+^ fraction was divided into CD27^+^ and CD27^−^ B cells. CD27^+^ cells were subdivided into marginal zone memory B cells (IgD^+^IgM^+^), class-switched memory B cells (IgD^−^IgM^−^), late memory B cells (CD38^+^IgM^+^) and plasmablasts (CD38^++^IgM^−^). CD27− cells were subdivided into innate B cells (IgD^−^IgM^−^), naïve B cells (IgD^+^IgM^+^), and transitional B cells (CD20^+^CD38^+^). CD20^+^ cells were subdivided into B1 cells (CD27^+^CD43^+^) and B2 cells (CD27^+^CD43^−^).


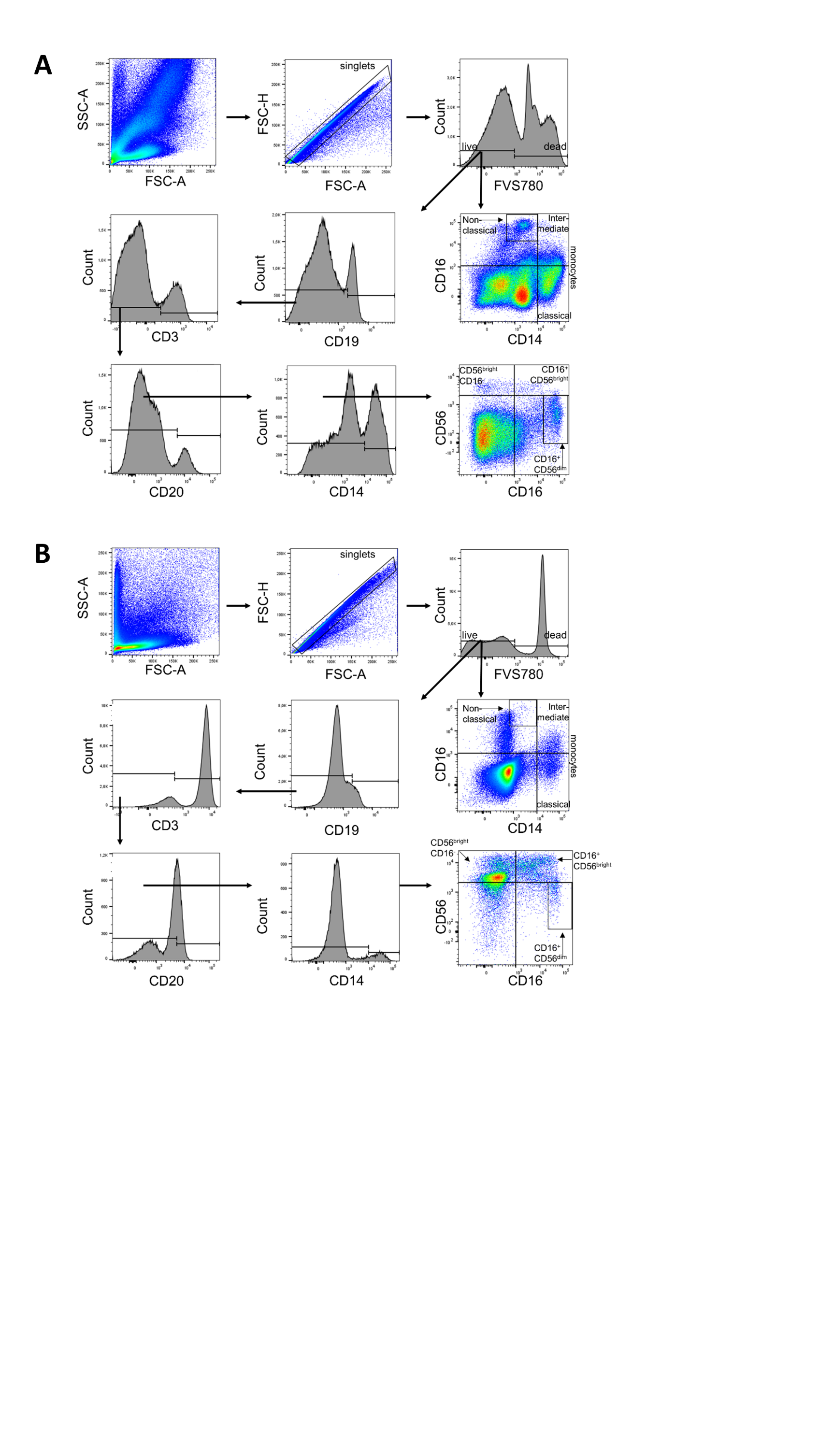


**Supplementary File 3:** Gating strategy (NK cells, monocytes)

Gating strategy of NK cells and monocytes subpopulations of a representative (A) PBMC and (B) PEC sample from a study participant. Analysis was performed using FlowJo (BD Biosciences). Gating was based first on morphological parameters (FSC-H/FSC-A) and FVS780 negative cells were considered as viable cells. On the one hand, within the viable cells were subdivided into monocytes subpopulations: Monocytes (CD14^+^), classical monocytes (CD14^+^CD16^-^), intermediate monocytes (CD14^+^CD16^+^) and non-classical monocytes (CD14^dim^CD16^+^). On the other hand, after exclusion of CD19^+^, CD3^+^, CD20^+^ and CD14^+^ cells from viable cells, NK cells subpopulations were determined as CD16^-^CD56^bright^ NK cells, CD16^+^CD56^bright^ NK cells and CD16^+^CD56^dim^ NK cells.

**Supplementary File 4:** Flow cytometry antibodies

| **marker** | **fluorophore** | **clone** | **vendor** | **catalog** | **RRID** |
| --- | --- | --- | --- | --- | --- |
|  |  |  |  |  |  |
| CD127 | Alexa-Fluor 647 | HIL-7R-M21 | BD | 558598 | AB_647113 |
| CD14 | BV421 | MΦP9 | BD | 563743 | AB_2744289 |
| CD16 | PE | 3G8 | BD | 555407 | AB_395807 |
| CD183 | BV480 | CXCR3 | BD | 746283 | AB_2743613 |
| CD19 | APC-R700 | HIB19 | BD | 564977 | AB_2744308 |
| CD194 | PE | 1G1 | BD | 551120 | AB_394054 |
| CD196 | APC-R700 | CCR6 | BD | 565173 | AB_2739092 |
| CD20 | BB700 | 2H7 | BD | 745889 | AB_2743319 |
| CD25 | BV421 | M-A251 | BD | 562442 | AB_11154578 |
| CD27 | BV650 | L128 | BD | 563228 | AB_2744352 |
| CD294 | BV650 | BM16 | BD | 740616 | AB_2740314 |
| CD3 | BV786 | SK7 | BD | 563800 | AB_2744384 |
| CD38 | FITC | HB7 | BD | 340927 | AB_400180 |
| CD4 | PE-CF594 | SK3 | BD | 566317 | AB_2739679 |
| CD43 | BV605 | 1G10 | BD | 563378 | AB_2738166 |
| CD45RO | BV605 | UCHL1 | BD | 562791 | AB_2744411 |
| CD56 | APC | B159 | BD | 555518 | AB_398601 |
| CD62L | BB700 | SK11 | BD | 745995 | AB_2743396 |
| CD8 | FITC | SK1 | BD | 347313 | AB_400279 |
| IgD | PE-CF594 | IA6-2 | BD | 562540 | AB_11153129 |
| IgM | BV480 | G20-127 | BD | 566146 | AB_2739544 |

**Supplementary File 5:** Cytokine levels (age)

| **cytokine** | **sample** | **age** | **median cytokine level [%]** | **p-value** |
| --- | --- | --- | --- | --- |
| GM-CSF | Plasma | < 12 months | 11.22 | 0.000008 |
|  |  | > 12 months | 70.38 |  |
|  | PE | < 12 months | 13.58 | 0.315 |
|  |  | > 12 months | 9.82 |  |
| IFN-γ | Plasma | < 12 months | 4.07 | 0.0001 |
|  |  | > 12 months | 64.98 |  |
|  | PE | < 12 months | 18.36 | 0.911 |
|  |  | > 12 months | 18.66 |  |
| IL-6 | Plasma | < 12 months | 30.14 | 0.385 |
|  |  | > 12 months | 20.55 |  |
|  | PE | < 12 months | 10000.00 | 0.233 |
|  |  | > 12 months | 8439.00 |  |
| IL-10 | Plasma | < 12 months | 22.46 | 0.079 |
|  |  | > 12 months | 50.08 |  |
|  | PE | < 12 months | 18.9 | 0,773 |
|  |  | > 12 months | 268.3 |  |
| IL-12p70 | Plasma | < 12 months | 1.91 | 0.0001 |
|  |  | > 12 months | 6.22 |  |
|  | PE | < 12 months | 1.22 | 0.893 |
|  |  | > 12 months | 1.17 |  |
| IL-13 | Plasma | < 12 months | 0.115 | 0.001 |
|  |  | > 12 months | 18.73 |  |
|  | PE | < 12 months | 1.23 | 0,272 |
|  |  | > 12 months | 6.76 |  |
| IL-17A | Plasma | < 12 months | 7.54 | 0.00004 |
|  |  | > 12 months | 36.2 |  |
|  | PE | < 12 months | 3.89 | 0.414 |
|  |  | > 12 months | 6.43 |  |
| IL-4 | Plasma | < 12 months | 6.01 | 0.001 |
|  |  | > 12 months | 119.6 |  |
|  | PE | < 12 months | 0.56 | 0.013 |
|  |  | > 12 months | 29.99 |  |
| IL-5 | Plasma | < 12 months | 2.35 | 0.0001 |
|  |  | > 12 months | 9.56 |  |
|  | PE | < 12 months | 13.05 | 0.229 |
|  |  | > 12 months | 7.24 |  |
| IL-7 | Plasma | < 12 months | 6.25 | 0.002 |
|  |  | > 12 months | 12.76 |  |
|  | PE | < 12 months | 22.06 | 0.683 |
|  |  | > 12 months | 17.77 |  |
| IL-8 | Plasma | < 12 months | 55.39 | 0.123 |
|  |  | > 12 months | 28.52 |  |
|  | PE | < 12 months | 1250 | 0.016 |
|  |  | > 12 months | 352.2 |  |
| TNF | Plasma | < 12 months | 19.41 | 0.031 |
|  |  | > 12 months | 13.82 |  |
|  | PE | < 12 months | 54.51 | 0.006 |
|  |  | > 12 months | 29.89 |  |

**Supplementary File 6:** Cytokine levels (CPB)

| **cytokine** | **sample** | **CPB** | **median cytokine level [%]** | **p-value** |
| --- | --- | --- | --- | --- |
| GM-CSF | Plasma | No CPB | 17.16 | 0.220 |
|  |  | CPB | 47.77 |  |
|  | PE | No CPB | 13.00 | 0.846 |
|  |  | CPB | 13.84 |  |
| IFN-γ | Plasma | No CPB | 13.75 | 0.218 |
|  |  | CPB | 56.23 |  |
|  | PE | No CPB | 20.48 | 0.551 |
|  |  | CPB | 22.15 |  |
| IL-6 | Plasma | No CPB | 35.16 | 0.586 |
|  |  | CPB | 28.64 |  |
|  | PE | No CPB | 10000.00 | 0.127 |
|  |  | CPB | 9219.00 |  |
| IL-10 | Plasma | No CPB | 30.20 | 0.165 |
|  |  | CPB | 55.73 |  |
|  | PE | No CPB | 232.80 | 0.517 |
|  |  | CPB | 220.40 |  |
| IL-12p70 | Plasma | No CPB | 3.05 | 0.225 |
|  |  | CPB | 4.61 |  |
|  | PE | No CPB | 1.02 | 0.476 |
|  |  | CPB | 1.67 |  |
| IL-13 | Plasma | No CPB | 0.44 | 0.012 |
|  |  | CPB | 20.37 |  |
|  | PE | No CPB | 0.35 | 0.020 |
|  |  | CPB | 12.44 |  |
| IL-17A | Plasma | No CPB | 9.41 | 0.178 |
|  |  | CPB | 29.31 |  |
|  | PE | No CPB | 6.70 | 0.484 |
|  |  | CPB | 5.17 |  |
| IL-4 | Plasma | No CPB | 16.05 | 0.077 |
|  |  | CPB | 101.80 |  |
|  | PE | No CPB | 2.24 | 0.017 |
|  |  | CPB | 103.40 |  |
| IL-5 | Plasma | No CPB | 3.04 | 0.070 |
|  |  | CPB | 8.97 |  |
|  | PE | No CPB | 12.31 | 0.938 |
|  |  | CPB | 11.50 |  |
| IL-7 | Plasma | No CPB | 7.63 | 0.241 |
|  |  | CPB | 11.53 |  |
|  | PE | No CPB | 25.23 | 0.794 |
|  |  | CPB | 21.45 |  |
| IL-8 | Plasma | No CPB | 57.26 | 0.816 |
|  |  | CPB | 31.88 |  |
|  | PE | No CPB | 843.90 | 0.936 |
|  |  | CPB | 1095.00 |  |
| TNF | Plasma | No CPB | 15.98 | 0.421 |
|  |  | CPB | 14.45 |  |
|  | PE | No CPB | 46.56 | 0.363 |
|  |  | CPB | 32.42 |  |

**Supplementary File 7:** Cytokine levels (sex)

| **cytokine** | **sample** | **sex** | **median cytokine level [%]** | **p-value** |
| --- | --- | --- | --- | --- |
| GM-CSF | Plasma | female | 35.98 | 0.59 |
|  |  | male | 54.12 |  |
|  | PE | female | 15.13 | 0.14 |
|  |  | male | 10.75 |  |
| IFN-γ | Plasma | female | 34.11 | 0.45 |
|  |  | male | 41.83 |  |
|  | PE | female | 24.48 | 0.73 |
|  |  | male | 18.36 |  |
| IL-6 | Plasma | female | 40.59 | 0.30 |
|  |  | male | 20.55 |  |
|  | PE | female | 10000.00 | 0.88 |
|  |  | male | 10000.00 |  |
| IL-10 | Plasma | female | 44.86 | 0.86 |
|  |  | male | 35.80 |  |
|  | PE | female | 197.30 | 0.93 |
|  |  | male | 258.00 |  |
| IL-12p70 | Plasma | female | 3.78 | 0.83 |
|  |  | male | 4.58 |  |
|  | PE | female | 1.22 | 0.56 |
|  |  | male | 1.17 |  |
| IL-13 | Plasma | female | 3.17 | >0.99 |
|  |  | male | 2.94 |  |
|  | PE | female | 10.11 | 0.52 |
|  |  | male | 3.22 |  |
| IL-17A | Plasma | female | 24.67 | 0.87 |
|  |  | male | 28.87 |  |
|  | PE | female | 6.43 | 0.54 |
|  |  | male | 4.63 |  |
| IL-4 | Plasma | female | 43.44 | 0.86 |
|  |  | male | 51.64 |  |
|  | PE | female | 17.35 | 0.65 |
|  |  | male | 11.83 |  |
| IL-5 | Plasma | female | 6.10 | 0.97 |
|  |  | male | 7.90 |  |
|  | PE | female | 22.74 | 0.26 |
|  |  | male | 7.94 |  |
| IL-7 | Plasma | female | 9.65 | 0.72 |
|  |  | male | 10.67 |  |
|  | PE | female | 17.68 | 0.65 |
|  |  | male | 21.45 |  |
| IL-8 | Plasma | female | 55.39 | 0.13 |
|  |  | male | 27.01 |  |
|  | PE | female | 1250.00 | 0.06 |
|  |  | male | 624.70 |  |
| TNF | Plasma | female | 15.55 | 0.42 |
|  |  | male | 14.38 |  |
|  | PE | female | 55.54 | 0.42 |
|  |  | male | 33.08 |  |
